# Are we aware how contaminated our mobile phones with nosocomial pathogens?

**DOI:** 10.1186/1476-0711-8-7

**Published:** 2009-03-06

**Authors:** Fatma Ulger, Saban Esen, Ahmet Dilek, Keramettin Yanik, Murat Gunaydin, Hakan Leblebicioglu

**Affiliations:** 1 Department of Anesthesiology and Reanimation, Faculty of Medicine, Ondokuz Mayis University, Kurupelit, 55139, Samsun, Turkey; 2 Department of Infectious Diseases and Clinical Microbiology, Faculty of Medicine, Ondokuz Mayis University, Kurupelit, 55139, Samsun, Turkey; 3 Department of Clinical Microbiology, Faculty of Medicine, Ondokuz Mayis University, Kurupelit, 55139, Samsun, Turkey

## Abstract

**Background:**

The objective of this study was to determine the contamination rate of the healthcare workers' (HCWs') mobile phones and hands in operating room and ICU. Microorganisms from HCWs' hands could be transferred to the surfaces of the mobile phones during their use.

**Methods:**

200 HCWs were screened; samples from the hands of 200 participants and 200 mobile phones were cultured.

**Results:**

In total, 94.5% of phones demonstrated evidence of bacterial contamination with different types of bacteria. The gram negative strains were isolated from mobile phones of 31.3% and the ceftazidime resistant strains from the hands were 39.5%. *S. aureus *strains isolated from mobile phones of 52% and those strains isolated from hands of 37.7% were methicillin resistant. Distributions of the isolated microorganisms from mobile phones were similar to hands isolates. Some mobile phones were contaminated with nosocomial important pathogens.

**Conclusion:**

These results showed that HCWs' hands and their mobile phones were contaminated with various types of microorganisms. Mobile phones used by HCWs in daily practice may be a source of nosocomial infections in hospitals.

## Background

Nosocomial infection is an important problem in all modern hospitals. As early as 1861 Semmelweis [1] demonstrated that bacteria were transmitted to the patients by the contaminated hands of healthcare workers. Hospital operating rooms (OR) and intensive care units (ICU) are the workplaces that need the highest hygiene standards, also the same requirements for the personnel working there and the equipment used by them. Some epidemiological studies have implicated environmental surfaces in the transmission of bacteria [2-4]. Mobile phones are widely used as nonmedical portable electronic devices and it is in close contact with the body. It is used for communication by health care workers in every location including OR and ICU. Studies do not include direct comparisons of transmission rates of bacteria from surfaces to hands. The risk of infection involved in using mobile phones in the OR and ICU has not yet been determined as there no cleaning guidelines available that meet hospital standards. However, the mobile phones are used routinely all day long but not cleaned properly, as health care workers' (HCW) may do not wash their hands as often as they should. The aim of the present study was to evaluate the role of mobile phones in relation to transmission of bacteria from the mobile phone to the healthcare workers' hands.

## Methods

The study was conducted in the eight beds of the mixed tertiary intensive care unit and 14 operating rooms. A total 200 staff – 15 senior, 79 assistant doctors, 38 nurses and 68 healthcare staff – were screened; cultures were subsequently obtained from the dominant hand of participants and their mobile phones at the same time. Gender, profession and duration of their profession, ring use, dominant hands of HCWs, routine cleaning of the mobile phones was recorded. A sterile swab moistened with sterile saline was rotated over the surface of both sides of mobile phones; second swab was rubbed over the entire ventral surface of the dominant hand (including ventral surfaces of the thumb and the fingers) of HCW's. The sampling of the dominant hand and mobile phone swabs (twice for hands and twice for mobile phones) were immediately streaked onto two plates that consist of blood agar supplemented with 5% defibrinated sheep blood and eosin methylene blue agar. Plates were incubated aerobically at 37°C for 48 h. Isolated microorganisms were identified using gram stain, colony counts, morphology, catalase and oxidase reaction and all isolates were allocated to the appropriate genera. For identification of gram negative bacteria VITEK 2 (bioMerieux, France) system was used. A slide coagulase test differentiated staphylococcal isolates into Staphylococcus aureus and coagulase-negative staphylococci (CoNS). Oxacillin sensitivity of the Staphylococci and ceftazidime sensitivity of the gram negative isolates were investigated by disk diffusion method according to Clinical Laboratory Standards (CLSI) criteria [5].

The protocol was approved by the ethical committee for human experimentation of Ondokuz Mayis University Faculty of Medicine and informed consent was obtained from the participants.

### Statistical analysis

Categorical variables were assessed by Chi square analysis. Non-categorical findings were assessed by the student t test or Man-Whitney U test. P values < 0.05 were considered significant. SPSS for Windows 13.0 software (SPSS Inc., Chicago, USA) was used for these analyses.

## Results

The rate of bacterial contamination of mobile phones is 94.5%. The isolated microorganisms from mobile phones and hands were similar (Table 1). Some of them are known to cause nosocomial infections. Hand contamination rates of HCWs and their personal mobile phones are shown in Table 2. It was found that 49.0% of phones grew one bacterial species, 34.0% two different species, 11.5% three or more different species and no bacterial growth were identified in 5.5% of phones.

**Table 1:** The types of bacteria isolated from phones and hands of HCW.

**Bacteria**		**Mobile phones (n = 200)**	**Hands of HCWs** **(n = 200)**
**Gram +**	Staphylococcus aureus	50	53
	
	Streptococcus spp.	12	18
	
	CoNS	181	193
	
	Enterococcus spp.	7	9

**Gram -**	Non-fermentative gram negatives	19	26
	
	Coliforms	15	12

**Other**	Moulds	20	19
	
	Yeasts	3	3

Total		307	333

**CoNS **(Coagulase negative staphylococci)

More than one type of bacterial growth were seen in some mobile phones

Those *S. aureus *strains isolated from mobile phones of 52.0% and those strains isolated from hands of 37.7% were methicillin resistant. The gram negative strains were isolated from mobile phones of 31.3% and the ceftazidime resistant strains from the hands were 39.5%. At the study period our nosocomial isolates at ICU were: 33.3% staphylococci, 21.4% non-fermentative gram negatives, 21.4% coliforms, 7.1% enterococci, 11.9% yeasts.

The rate of routine cleaning of HCW's mobile phones was 10. 5%, which means 89.5% of the participants never cleaned their mobile phones. Although the assistant doctors' phones have higher colony count there was no significant difference in the rates of specific types of bacterial growth and colony counts isolated on all groups' mobile phones (Table 2).

**Table 2:** Hand contamination rate of HCWs and colony count with or without ring

**Profession**	**N (Mean ± SD)**	**Ring using staff's Mobile phone** **(Mean ± SD)**	**Non ring using staff's Mobile phone (Mean ± SD)**
**Assistant doctor**	79 (19.0 ± 35.8)	13.1 ± 36.4	20 ± 35.8

Healthcare personnel*	68 (18.4 ± 41.3)	24.2 ± 57.5	15.05 ± 26.3

**Senior doctor**	15 (12.8 ± 15.1)	17.0 ± 19.3	9.42 ± 10.0

**Nurse**	38 (10.7 ± 28.7)	46.4 ± 86.1	6.7 ± 8.03

Healthcare personnel* (Nurse, physiotherapist, student nurse, etc.)

25.5% of the entire study population had one or more rings. The mean colony count was higher in ring using staff's phones but there was no significant difference between rate of contamination and colony count (Table 2) (p > 0.05).

## Discussion

In this study, the use of mobile phones by HCWs working in OR and ICU not only demonstrated a high contamination rate with bacteria but also more importantly contamination with nosocomial pathogens. The possibility transmissions of nosocomial pathogens by electronic devices such as personal digital assistants, handheld computers, and bedside applications were previously reported and some of them were epidemiologically important drug-resistant pathogens [6,7]. Isaacs et al. [6] showed that the main growth was of coagulase-negative staphylococci from 25 keyboards. Two keyboards grew *S. aureus*, both of which samples were susceptible to methicillin/flucloxacillin. Neely et al. [8] also identified nosocomial *A. baumannii *infection on keyboards as a reservoir in burn units and ICUs. Butz et al. [9] stated that immobile phones might carry pathogens as well; stationary phones in a daycare facility were contaminated with rotavirus Rusin et al. [10] documented that hand-to-mouth transfer of microbes after handling contaminated fomites during casual activities. Singh et al. [11] reported that over 47% of immobile phones were contaminated with pathogenic microbes.

These results suggested that close contact objects that were contaminated could serve as reservoirs of bacteria where could be easily transmitted from the mobile phone to the HCWs' hands. During every phone call the mobile phones come into close contact with strongly contaminated human body areas with hands to hands and hands to other areas (mouth, nose, ears). Herein mobile phones are particularly problematic when compared to immobile devices and it may facilitate transmission of bacterial isolates from patient to patient in wards or hospitals.

Some authors [12,13] showed that HCWs' mobile phones were contaminated with nosocomial pathogens. The result of our study demonstrated cross transmission of bacteria between HCWs' dominant hands and one third of mobile phones. Gram negative bacteria are very important nosocomial pathogens and HCWs' mobile phones were carried ceftazidime resistant Gram negative isolates and half of *S. aureus *isolates were resistant to methicillin. However, this study was carried on a limited scale as no molecular tests were conducted for showing clonal relation.

Our study demonstrated that the isolated microorganisms from hands and phones were similar. It is clear that it is not possible to estimate the level of bacterial contamination with one sampling technique. Borer at al. [12] observed that there were contaminations of hands and mobile phones only in 10% of their staff who were sampled for once. The present study was nevertheless similarly planned; in this study contamination rate of the mobile phones was 94.5% for one sampling. Since no warning has been given for cleaning mobile phones to meet hospital standards, the same rates and composition of contamination of mobile phones could be risky when carried outside the hospital environment. Limitation or crackdown of these items would be unpractical strategies for preventing nosocomial transmission, because mobile phones are used by the personnel both in private communication and emergency situations in ICU so; cross-transmissions between hands to mobile phones were assessed. Although it seems impossible, in the light of all these findings, we should be aware of limiting the mobile phone usage as it has high risk for spreading infections.

According to these results it is obvious that, the training of healthcare personnel about strict infection control procedure, hand hygiene, environmental disinfection, and eventually, optimum disinfection methods are of great importance. Otherwise, the potential benefit of using mobile phones by the personnel for private communication or emergency situations in ICU or OR would change into this means of communication detrimental to hospital hygiene. Therefore, near the hand hygiene, cleaning of these devices should be kept in mind. Prevention of contamination risk of nosocomial pathogens and infections stands out as problem that must be weighed in mind.

Developing active preventive strategies like routine decontamination of mobile phones with alcohol containing disinfectant materials might reduce cross-infection. Another way of reducing bacterial contaminations on mobile phones might be the use of antimicrobial additive materials. We could easily avoid spreading bacterial infections just by using regular cleansing agents and rearranging our environment. In the future mobile phones could be produced by using protective material against the bacterial contamination.

## Competing interests

The authors declare that they have no competing interests.

## Authors' contributions

FU carried out the design of the study, participated in the sequence alignment and drafted the manuscript. SE participated in the design of the study and performed the statistical analysis. AD participated in the sequence alignment and helped to draft the manuscript. MG and KY carried out the microbiological procedures. HL conceived of the study, and participated in its design and coordination. All authors have read and approved the final manuscript.
