# Correction: Are we aware how contaminated our mobile phones with nosocomial pathogens?

**DOI:** 10.1186/1476-0711-8-31

**Published:** 2009-11-13

**Authors:** Fatma Ulger, Saban Esen, Ahmet Dilek, Keramettin Yanik, Murat Gunaydin, Hakan Leblebicioglu

**Affiliations:** 1 Department of Anesthesiology and Reanimation, Faculty of Medicine, Ondokuz Mayis University, Kurupelit, 55139, Samsun, Turkey; 2 Department of Infectious Diseases and Clinical Microbiology, Faculty of Medicine, Ondokuz Mayis University, Kurupelit, 55139, Samsun, Turkey; 3 Department of Clinical Microbiology, Faculty of Medicine, Ondokuz Mayis University, Kurupelit, 55139, Samsun, Turkey

## Correction

The following reference, "Jeske HC, Tiefenthaler W, Hohlrieder M, Hinterberger G, Benzer A. Bacterial contamination of anaesthetists' hands by personal mobile phone and fixed phone use in the operating theatre. Anaesthesia 2007, 62(9):904-6." was omitted mistakenly at the background section and should be added to literature sited section of this manuscript [1]. The author regrets for the oversights and thanks to editors for giving opportunity to both cite and give proper credit to Dr. Jeske's study.

The correct reference and paragraph are printed below:

## Background

Nosocomial infection is an important problem in all modern hospitals. As early as 1861 Semmelweis [2] demonstrated that bacteria were transmitted to the patients by the contaminated hands of healthcare workers. Hospital operating rooms (OR) and intensive care units (ICU) are the workplaces that need the highest hygiene standards, also the same requirements for the personnel working there and the equipment used by them [3]. Some epidemiological studies have implicated environmental surfaces in the transmission of bacteria [4-6]. Mobile phones are widely used as nonmedical portable electronic devices and it is in close contact with the body It is used for communication by health care workers in every location including OR and ICU. Studies do not include direct comparisons of transmission rates of bacteria from surfaces to hands. The risk of infection involved in using mobile phones in the OR and ICU has not yet been determined as there no cleaning guidelines available that meet hospital standards [3]. However, the mobile phones are used routinely all day long but not cleaned properly, as health care workers' (HCW) may do not wash their hands as often as they should. The aim of the present study was to evaluate the role of mobile phones in relation to transmission of bacteria from the mobile phone to the healthcare workers' hands.
